# Influence of stroke infarct location on quality of life assessed in a multivariate lesion-symptom mapping study

**DOI:** 10.1038/s41598-021-92865-x

**Published:** 2021-06-29

**Authors:** Alina Königsberg, Andrew T. DeMarco, Carola Mayer, Anke Wouters, Eckhard Schlemm, Martin Ebinger, Tae-Hee Cho, Matthias Endres, Jochen B. Fiebach, Jens Fiehler, Ivana Galinovic, Josep Puig, Vincent Thijs, Robin Lemmens, Keith W. Muir, Norbert Nighoghossian, Salvador Pedraza, Claus Z. Simonsen, Christian Gerloff, Götz Thomalla, Bastian Cheng

**Affiliations:** 1 Klinik und Poliklinik für Neurologie, Kopf- und Neurozentrum, University Medical Center Hamburg-Eppendorf, Martinistr. 52, 20246 Hamburg, Germany; 2 Department of Rehabilitation Medicine, Georgetown University, Washington, DC USA; 3 Department of Neurology, University Hospitals Leuven, Herestraat 49, 3000 Leuven, Belgium; 4 Department of Neurosciences, Experimental Neurology, KU Leuven – University of Leuven, Oude Markt 13, Bus 5005, 3000 Leuven, Belgium; 5 VIB, Center for Brain & Disease Research, Laboratory of Neurobiology, Campus Gasthuisberg, Herestraat 49, Bus 602, 3000 Leuven, Belgium; 6 Centrum für Schlaganfallforschung Berlin (CSB), Charité - Universitätsmedizin Berlin, Campus Mitte, Charitéplatz 1, 10117 Berlin, Germany; 7 Neurologie der Rehaklinik Medical Park Humboldtmühle, An der Mühle 2-9, 13507 Berlin, Germany; 8 Department of Stroke Medicine, Université Claude Bernard Lyon 1, CREATIS CNRS UMR 5220-INSERM U1206, INSA-Lyon, Hospices Civils de Lyon, Lyon, France; 9 Klinik und Hochschulambulanz für Neurologie, Charité-Universitätsmedizin Berlin, Campus Mitte, Charitéplatz 1, 10117 Berlin, Germany; 10 Department of Diagnostic and Interventional Neuroradiology, University Medical Center Hamburg-Eppendorf, Martinistr. 52, 20246 Hamburg, Germany; 11 Department of Radiology, Institut de Diagnostic per la Image (IDI), Hospital Dr Josep Trueta, Institut d’Investigació Biomèdica de Girona (IDIBGI), Parc Hospitalari Martí i Julià de Salt - Edifici M2, 17190 Salt, Girona Spain; 12 Stroke Division, Florey Institute of Neuroscience and Mental Health, University of Melbourne, 245 Burgundy Street, HeidelbergVictoria, VIC 3084 Australia; 13 Department of Neurology, Austin Health, 145 Studley Road, Heidelberg, VIC 3084 Australia; 14 Institute of Neuroscience & Psychology, University of Glasgow, Queen Elizabeth University Hospital, Glasgow, UK; 15 Department of Neurology, Aarhus University Hospital, 8200 Aarhus, Denmark; 16 Neurology, Amsterdam University Medical Centers, AMC, Amsterdam, The Netherlands

**Keywords:** Neuroscience, Medical research, Neurology

## Abstract

Stroke has a deleterious impact on quality of life. However, it is less well known if stroke lesions in different brain regions are associated with reduced quality of life (QoL). We therefore investigated this association by multivariate lesion-symptom mapping. We analyzed magnetic resonance imaging and clinical data from the WAKE-UP trial. European Quality of Life 5 Dimensions (EQ-5D) 3 level questionnaires were completed 90 days after stroke. Lesion symptom mapping was performed using a multivariate machine learning algorithm (support vector regression) based on stroke lesions 22–36 h after stroke. Brain regions with significant associations were explored in reference to white matter tracts. Of 503 randomized patients, 329 were included in the analysis (mean age 65.4 years, SD 11.5; median NIHSS = 6, IQR 4–9; median EQ-5D score 90 days after stroke 1, IQR 0–4, median lesion volume 3.3 ml, IQR 1.1–16.9 ml). After controlling for lesion volume, significant associations between lesions and EQ-5D score were detected for the right putamen, and internal capsules of both hemispheres. Multivariate lesion inference analysis revealed an association between injuries of the cortico-spinal tracts with worse self-reported quality of life 90 days after stroke in comparably small stroke lesions, extending previous reports of the association of striato-capsular lesions with worse functional outcome. Our findings are of value to identify patients at risk of impaired QoL after stroke.

## Introduction

Ischemic stroke affects health-related quality of life of patients in various domains beyond impairments captured by scales such as the National Institutes of Health Stroke Scale (NIHSS) or modified Rankin Scale (mRS)^1,2^. In recent years, the importance of patient reported outcome measures (PROMs) in stroke care and research has been addressed, as these capture the patient’s own perspective on quality of life and are therefore of relevance to promote shared decision-making, facilitate personalized health care and ultimately improve patients well-being after stroke^3,4^. Several questionnaires and scores are available to operationalize quality of life (QoL) after stroke. The European Quality of Life 5 Dimensions (EQ-5D) represents one of the most widely used instruments in clinical studies and population surveys, and includes five key dimensions of self-reported QoL: mobility, self-care, usual activities, pain or discomfort and anxiety or depression^5^.

Stroke lesion location exerts a major impact on the degree of clinical deficits, recovery and functional outcome^6,7^. Anatomical localization of stroke lesions in strategic brain areas results in proportionally higher scores on the mRS and is therefore of interest to predict stroke severity beyond traditional imaging markers such as lesion volume^6^. In addition, mapping clinical scales to (eloquent) brain areas may improve understanding of the underlying structure–function relationship. With regard to the EQ-5D as a short form of PROM, associations between stroke lesion location and QoL measures remain largely unexplored. We therefore investigated the impact of stroke lesion location on the EQ-5D based on clinical and imaging data from the WAKE-UP trial, a multicenter-randomized, double-blind, placebo-controlled trial of MRI-based intravenous thrombolysis in patients with unknown onset stroke. We applied a voxel-wise, multivariate, lesion-symptom mapping (LSM) technique based on a supervised machine learning algorithm (support vector regression, SVR) to account for nuisance variables and lesion patterns beyond single voxels that cannot be resolved by traditional, mass-univariate models^8^. We hypothesized that by this approach, we would identify brain areas responsible for sensory, motor and affective functions that feature prominently in the EQ-5D.

## Results

### Descriptive statistics

Of 503 patients randomized in WAKE-UP, EQ-5D scores were available for 452 patients. Of patients with missing EQ-5D (N = 51), 13 died and for N = 38, EQ-5D was not available. Stroke lesion segmentation and registration to MNI was successful in 349 (of 452) patients on follow-up imaging 22–36 h after randomization. Reasons for missing data (n = 103) were: missing imaging (either FLAIR or DWI) in 21 patients, hemorrhagic transformation (n = 23), CT imaging on follow-up (n = 20) and insufficient imaging quality (n = 39). Mean age of the 349 included patients was 65.4 years (SD 11.5), 228 (65.3%) patients were male, median NIHSS score was 6 (IQR 4–9). 90 days after stroke, median EQ-5D score at 90 days was 1 (IQR 0–4) and median mRS score 2 (IQR 1–3). Median DWI stroke lesion volume was 3.3 ml (IQR 1.1 ml–16.9 ml, mean 17.3 ± 33.3 ml, minimum 0.02 ml, maximum 233 ml). Stroke lesions were located in the left hemisphere in 190 patients, right hemisphere in 136 and in both hemispheres in 23.

**Figure 1:**
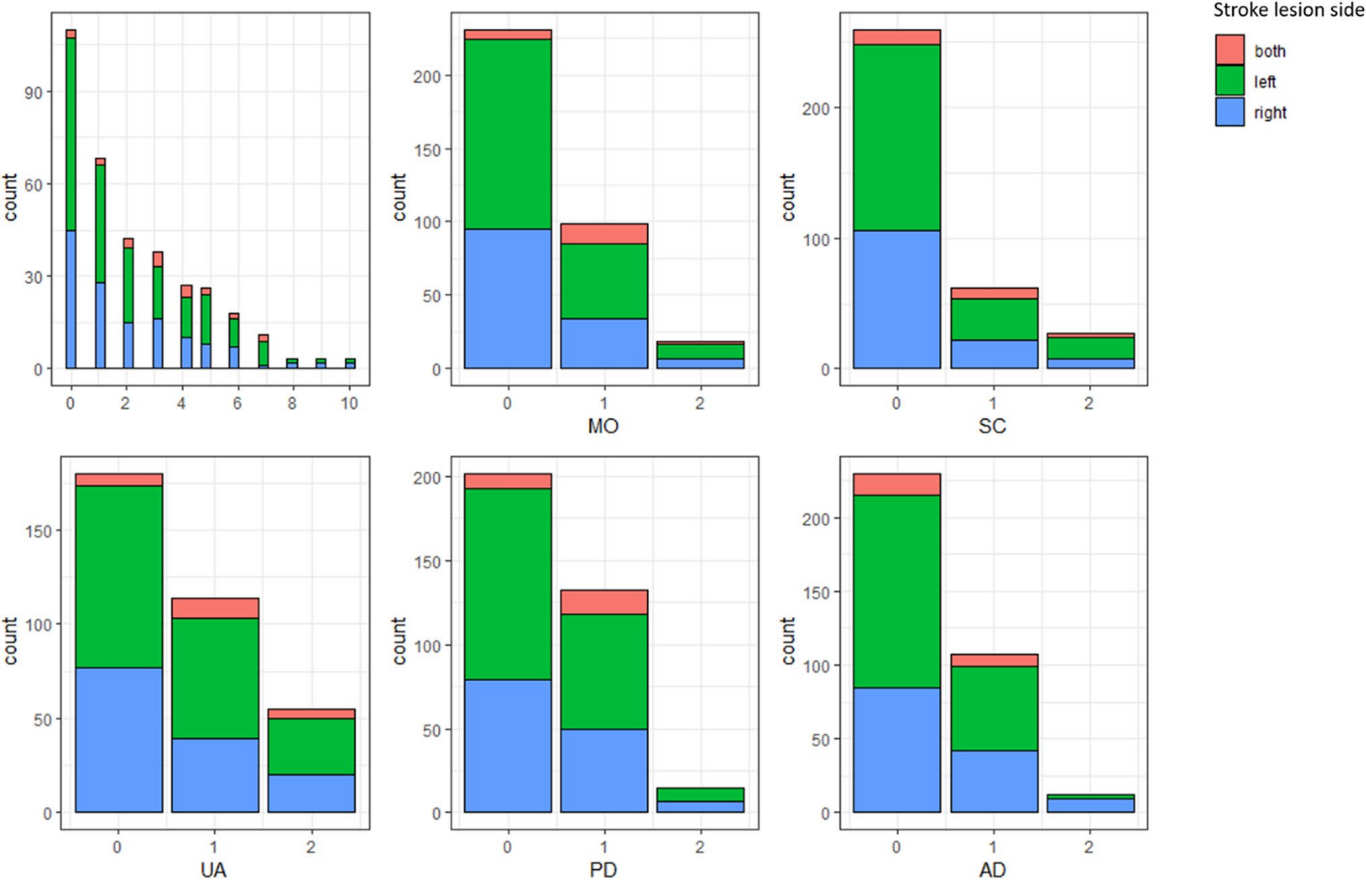
Histograms demonstrating the distribution of EQ-5D sum scores and subscores. Higher scores relate to lower self-reported measures of quality of life. Affected hemisphere is indicated by color (both hemispheres = red, left hemisphere = green, right hemisphere = blue).

**Figure 2:**
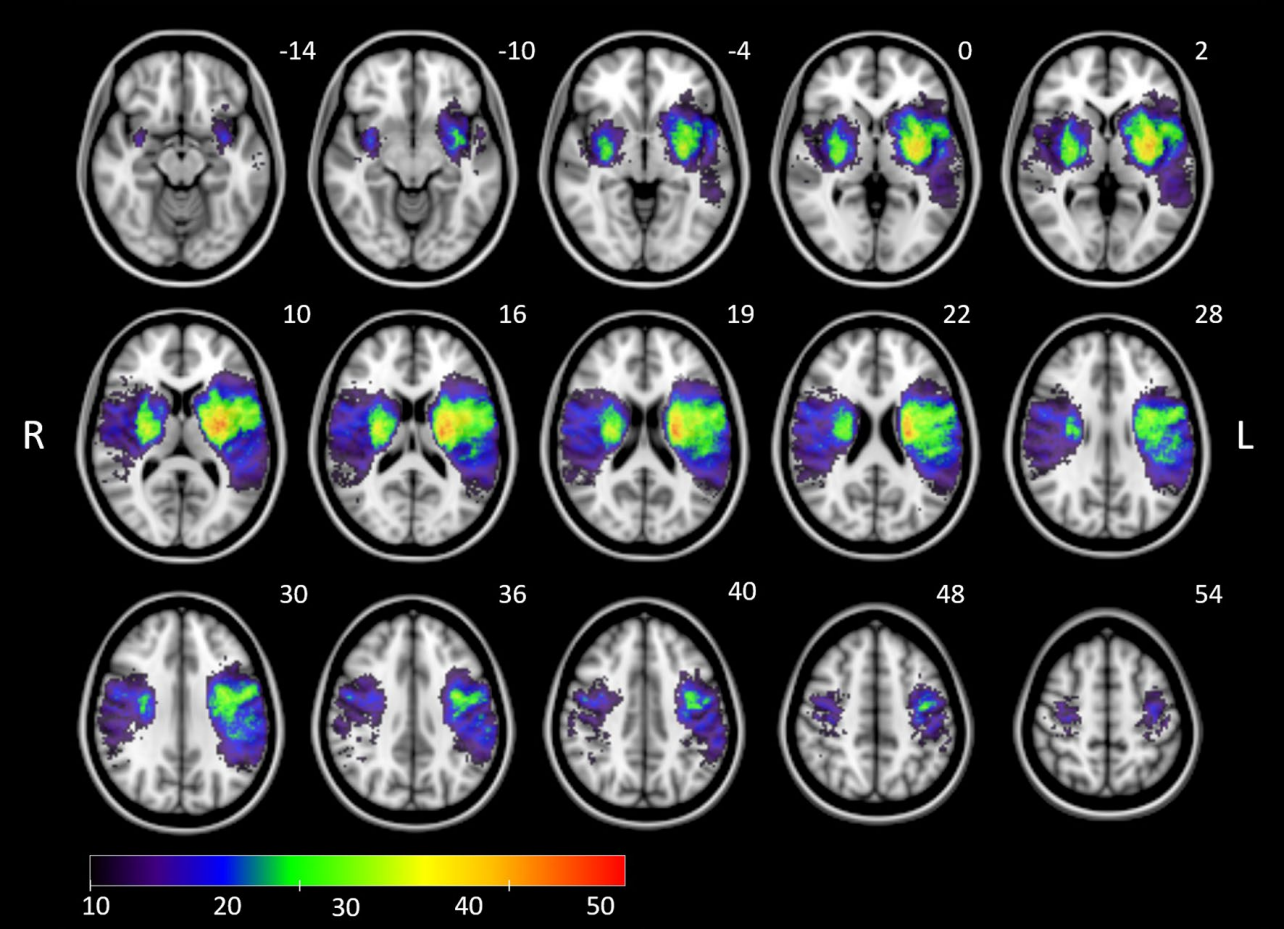
Overlay lesion plot of stroke lesions from all patients (n = 329) in radiological convention mapped onto the Montreal Neurological Institute (MNI) template. Overlays are thresholded to show lesions present in at least 10 individuals as included in the lesion-symptom mapping analysis. The color bar indicates lesion frequency across all patients. MNI coordinates of each transverse section (z axis) are indicated in white numbers above the slices.

Figure 1 illustrates the distributions of the EQ-5D sum score and individual subscores stratified by affected hemisphere. Whereas the majority of patients reported few complaints in individual subscores (0 points), moderate or severe problems (1 or 2 points) were reported for example in up to 49% of patients regarding the performance of usual activities (EQ-5D subscore 3). There was a significant correlation between higher EQ-5D scores 90 days after stroke with higher mRS scores (Spearman r = 0.742, p < 0.001). 20 patients were excluded from SVR-LSM due to non-overlap of lesioned voxels with the minimum lesion cutoff mask. Figure 2 displays the distribution of stroke lesions of patients that were finally included by SVR-LSM (n = 329) overlaid on an MNI standard space template.

**Figure 3:**
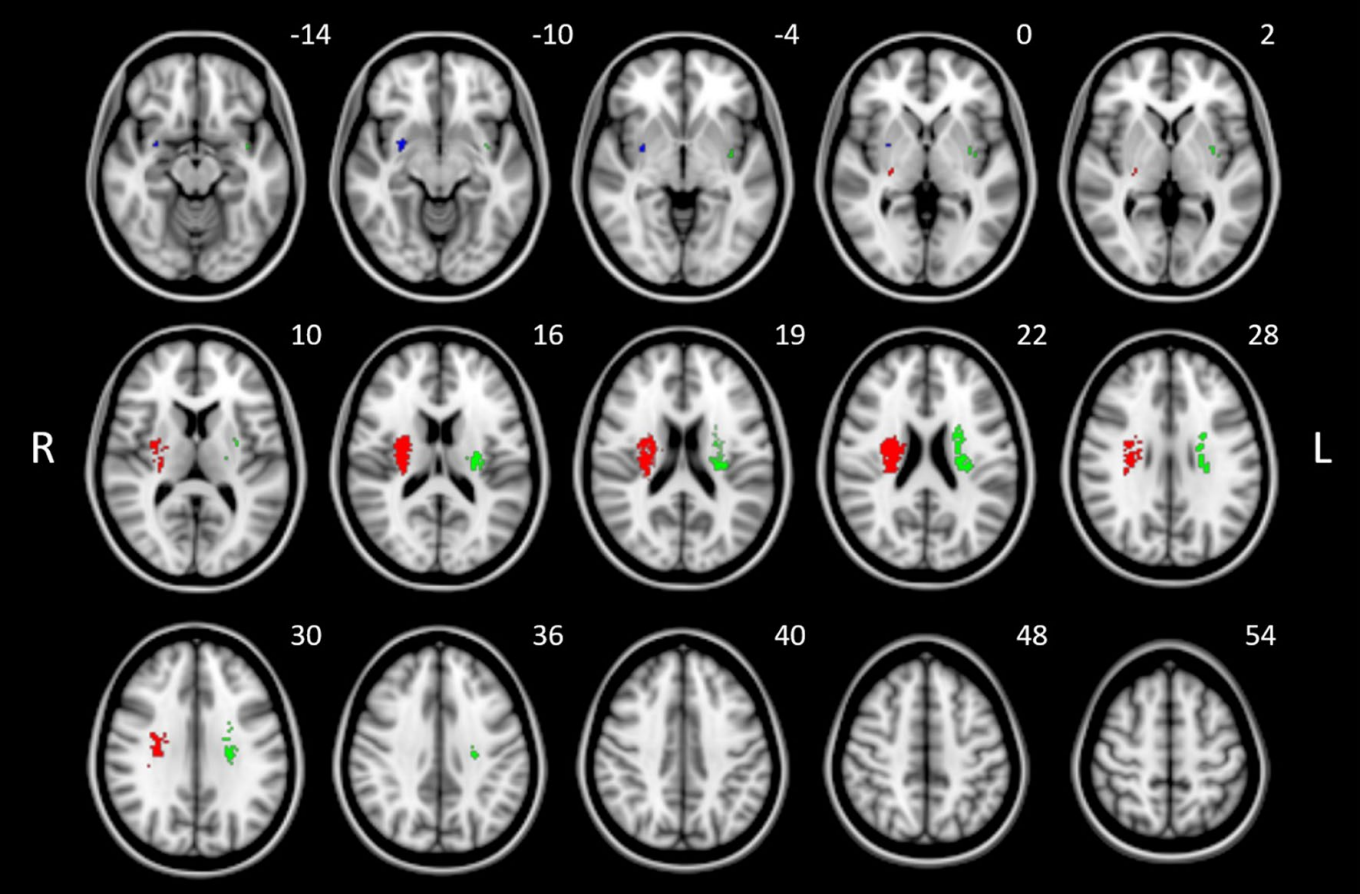
Results of SVR lesion-symptom mapping for the EQ-5D sum scores illustrated on a brain template in MNI standard space oriented in radiological convention. Three clusters with N > 50 significant voxels after permutation based on a threshold of p < 0.005 are shown. Clusters are color-coded to identify p-values after cluster-wise FWE correction (p = 0.036 [red]; p = 0.062 [green]; p = 0.298 [blue]). See also Table 1 for statistical and anatomical details. MNI coordinates of each transverse section (z axis) are shown.

SVR LSM detected three clusters containing more than 50 lesioned voxels associated with higher sum scores of EQ-5D, i.e., worse self-reported QoL (see Table 1). Of those, one cluster reached the FWE correction p-value (p < 0.05) and was located in the right corticospinal tract at the level of the internal capsule and corona radiata. As illustrated in Fig. 3, the remaining two clusters with > 50 voxels were located at the putamen, corticospinal tract and uncinate fasciculus. Details are listed in Table 1. Model quality measures were implemented as previously described^9^ and indicated sufficient prediction accuracy and pattern reproducibility with values comparable to previous analogous applications of SVR-LSM methods (see Supplemental Table S2 and Fig. S1)^10^. Analysis of lesion cluster locations in reference to white matter fibers by the BCB (Brain Connectivity and Behavior) Toolkit (Foulon et al.^11^; http://www.toolkit.bcblab.com) revealed a high certainty of overlap voxels associated with high EQ5D scores with the corticospinal tract, pontine and thalamic projections and superior longitudinal fasciculus, among others (Table 2).

**Table 1:** Results of SVR lesion-symptom mapping for the EQ-5D sum scores.

Cluster number and color (Fig. 3)	Cluster wise FWE corrected p-value	Number of voxels	Anatomical location	MNI center coordinates (X, Y, Z)
1, Red	0.0363*	717	Right corticospinal tract	27.9; − 13; 20.1
2, Green	0.0624	444	Left corticospinal tract	− 26.4; − 12.8; 20.7
3, Blue	0.2981	53	Right putamen Right uncinate fascicle	29.7; − 0.2; − 8.2

Clusters with N > 50 significant voxels after permutation based on a voxel-wise threshold of p < 0.005 are shown. Results are listed by increasing cluster wise FWE p-value. MNI coordinates are given at the center location of each cluster and the anatomical regions.

*p < 0.05 resulting from FWE correction.

**Table 2:** Certainty of non-zero overlap between the union of clusters, if they contained > 50 voxels and were identified by SVR-LSM for the EQ-5D sum score (cf. Table 1), and anatomically pre-defined white matter tracts (implemented in the Tractotron software, standalone version of the BCB Toolkit: Foulon et al.^11^; http://www.toolkit.bcblab.com).

Tract	Right hemisphere (%)	Left hemisphere (%)
Corticospinal tract	100	100
Fronto-striatal tract	100	100
Pontine projections	100	100
Superior longitudinal fasciculus II	97	98
Superior longitudinal fasciculus III	100	99
Anterior thalamic projections	96	98
Frontal aslant tract	–	100
Corpus callosum	90	100
Inferior fronto-occipital fasciculus	100	100
Uncinate fasciculus	100	98
Anterior commissure	96	–
Fronto-insular tract 4	96	–
Fronto-insular tract 5	100	–

Displayed are only tracts which contain, with a probability > 90%, at least one voxel associated with higher EQ-5D scores.

## Discussion

The aim of this study was to explore lesion-symptom relationships for self-reported quality of life after stroke. As a main result, we demonstrate the association of higher EQ-5D values 90 days after stroke with lesions located in deep grey matter and white matter structures involved in motor functions, such as the basal ganglia and cortico-spinal tracts of both hemispheres.

Up to now, few studies have investigated the association between lesion location and self-reported measures of QoL after stroke. Grouping lesions by imaging-informed etiology, a previous study reported hemorrhages, territorial or supratentorial strokes to be associated with subsequent lower QoL 6 months after stroke compared to ischemic, infratentorial or lacunar strokes^12^. Except for domains involving speech, there was no clear relationship between stroke laterality and QoL^12^. In contrast, an analysis investigating 229 patients with minor stroke symptoms (NIHSS ≤ 5), found that stroke lesions located in the brainstem and subcortical strokes were associated with lower self-reported QoL compared to stroke lesions involving the cerebral cortex^13^. Our findings extend observations from these studies by providing high-resolution anatomical locations of lesions impacting quality of life measured by the EQ-5D in a large cohort of acute stroke patients.

In our main analysis, worse QoL after stroke measured by EQ-5D was primarily associated with stroke lesions located at the corticospinal tract at the level of the internal capsule (Table 1, Fig. 3). Although the cluster located at the left cortico-spinal tract did not achieve full FWE correction for cluster extent (p = 0.062), we would not consider this as a false-positive result given the close proximity to the predefined statistical threshold (p < 0.05), anatomical plausibility, cluster size and the cluster’s survival of voxel-wise permutation testing performed by SVR-LSM. These findings are plausible given the fact that motor functions influence at least three of five domains captured in the EQ-5D, specifically mobility, self-care and usual daily activities^5^. In line with this observation, high scores on common stroke severity scales capturing impairment of motor function such as the NIHSS and mRS have been shown to contribute significantly to reduced self-reported quality of life measured by the EQ-5D and similar instruments^14–17^. In our study, worse clinical outcome as measured by the mRS was also associated with lower QoL as measured by the EQ-5D. From the perspective of lesion-symptom relationship inference, higher mRS scores 3 months after stroke have previously been shown to be associated with lesions located in the corticospinal tract similar to our current findings from mapping the EQ-5D^7^.

In addition to mapping anatomical location, we explored the relevance of brain areas identified by lesion-symptom inference leading to lower EQ-5D in reference to an atlas of white matter fiber tracts (Table 2). As expected, this showed involvement of white matter tracts facilitating motor functions such as the corticospinal and fronto-striatal tract as well as pontine projections. However, this indirect analysis also revealed a potential involvement of various fiber bundles traversing inter- and intra-hemispheric brain regions such as the superior longitudinal fasciculus or corpus callosum. These findings motivate future investigations of structural disconnection and suggest more complex contributions of structural connectivity beyond classical motor pathways contributing to quality of live after stroke.

We assessed self-reported QoL using the EQ-5D questionnaire, which is a prominent and widely applied scales in stroke care^14,18–20^. However, questionnaires that capture aspects of QoL in more detail then the E5QD are available specifically in stroke populations like the Stroke Impact Scale (SIS) and Stroke-Specific Quality of Life Scale (SS-QoL)^19^. Although our findings are not directly generalizable to results obtained by these more specific instruments, physical disability features prominently in almost all patient-reported measures and we would therefore argue that our findings are valid regarding the self-perceived and -reported information on quality of life in our patients^3^.

Strengths of our study are the inclusion of clinical data from patients from a large, prospective clinical stroke trial, definition of lesions based on MRI and the use of an innovative multivariate lesion-symptom mapping approach that is superior to traditional mass-univariate LSM methods.

In terms of limitations, PROMs generally tend to under-represent severely affected patients and patients with deficits that restrain them from participating in a questionnaire, also there might be a bias towards patients presenting with motor deficits and clinically not-apparent or improved language and visuo-spatial deficits. Unavailable questionnaires in this study might have influenced the results. As three out of five categories of the EQ-5D relate to motor functions, other aspects of QoL might be rather underrepresented by the questionnaire. As an additional limitation, lesion distribution in WAKE-UP, similar to other stroke trials, may have biased our results towards brain functions located in brain areas supplied by the middle cerebral artery (MCA). Therefore, lesion-symptom inference for QoL regarding stroke lesions located in brain areas such as the frontal cortex or areas supplied by the posterior arterial circulations, have to be explored in future studies. Moreover, the median lesion size was rather small compared to a “traditional” stroke patient cohort, the findings of this study can hence not be directly transferred to larger sized stroke lesions. Clinical factors like hemiparesis were not included in the analysis, therefore a possible influence of these could not be commented on.

## Summary

In summary, multivariate lesion inference analysis revealed an association of comparably small acute stroke lesions in the internal capsule with worse self-reported quality of life 90 days after stroke. These findings extend previous reports of the association of striato-capsular lesions with worse functional outcome of stroke. Our findings are of value to identify stroke patients at risk of impaired QoL and illustrate dependencies of the EQ-5D as a popular PROM on anatomical localization of brain injury.

## Methods

### Study design

We analyzed clinical and imaging data from the WAKE-UP trial (ClinicalTrials.gov number, NCT01525290), a multicenter, randomized, double-blind, placebo-controlled trial of MRI-based intravenous thrombolysis in patients with unknown onset stroke. The detailed trial protocol and the results of the WAKE-UP trial have been published previously^21^. Briefly summarized, patients presenting with signal change on Diffusion weighted imaging (DWI) but no visible abnormality on T2-fluid-attenuated inversion recovery (FLAIR) were selected on the basis of probable stroke onset in the preceding 4.5 h and randomized to receiving alteplase or placebo. Written informed consent was provided according to national and local regulations by patients or their legal representatives with an exception from explicit informed consent in emergency circumstances in some countries. All methods were carried out in accordance with relevant guidelines and regulations. All experimental protocols were approved by the ethics committee of the Hamburg chamber of physicians and Federal Institute for Drugs and Medical Devices (BfArM) as regulatory authority.

For this secondary analysis, we selected only patients included in the WAKE-UP trial for whom both MRI data and a completed European Quality of Life 5 Dimensions (EQ-5D) 3 level form 90 days after stroke were available. Imaging data were collected from MRI performed after randomization, 22–36 h after admission. Due to incorrect segmentation in the presence of larger areas with signal loss, we excluded patients with MR images of insufficient quality and those with hemorrhagic transformation of stroke. We collected clinical data from WAKE-UP, including patient age at symptom onset, sex, randomization result (alteplase or placebo), NIHSS on admission, and mRS score 90 days after stroke.

Self-reported quality of life was assessed 90 days after stroke via the EQ-5D-3L questionnaire^5^. It consists of five subscores (mobility, self-care, usual activities, pain/discomfort and anxiety/depression), each featuring three levels of severity of impairment (Supplementary Table S1). Individual subscores were added to calculate the EQ-5D sum scores.

Descriptive statistics of clinical and imaging data are provided as mean, median and standard deviation (SD) or interquartile range (IQR), where appropriate. A Spearmann rank-order correlation was applied to assess the association between mRS and EQ-5D at 90 days after stroke. Statistical significance was defined as p < 0.05. The statistical analysis was performed in R (v3.1.4).

### Image analysis

Stroke lesions were segmented and lesion volumes quantified as described previously^22^. In summary, imaging data were analyzed by dedicated software developed for the WAKE-UP trial (Stroke Quantification Tool, SONIA) performing registration and semi-automated stroke lesion segmentation based on an apparent diffusion coefficient (ADC) threshold of 620 mm^2^/s. After quality control of the segmentation and manual correction where necessary, lesion volumes were calculated on binary lesion maps in native space. Afterwards, lesion masks were transformed to Montreal Neurological Institute (MNI) space by linear and non-linear registrations based on FLAIR data^23^. All lesions masked were checked for correct segmentation and registration into MNI-space by two raters experienced in stroke MR imaging (A. K., B. C.). Imaging data with erroneous registration were excluded from analysis.

### Lesion symptom mapping

Multivariate lesion-symptom mapping was conducted using support vector regression (SVR), a supervised learning algorithm that extends support vector machines and allows for regression involving continuous variables^24^. SVR-LSM can be applied for lesion-symptom inference with superior sensitivity and specificity compared to traditional mass-univariate lesion-symptom methods, taking into account lesion patterns distributed over separate voxels^8^. SVR was conducted using a publicly available SVR lesion symptom mapping toolbox (SVR-LSM, available at https://github.com/atdemarco/svrlsmgui) applying functionalities of the Statistics and Machine Learning Toolbox within MATLAB (MATLAB 2019b, The MathWorks, Inc., Natick, Massachusetts, United States)^25^. In summary, SVR creates regression models assigning a “feature weight” to each voxel based on its lesion status and a behavioral variable. Multiple random permutations of the SVR on behavioral data are then performed to reveal statistically significant associations based on pre-defined statistical thresholds. For our analysis, only voxels with a minimum of 10 overlapping lesions were included for statistical testing in line with previous applications in similar datasets. We conducted the SVR-LSM analysis for the sum score of EQ-5D with higher values signifying lower quality of life. The SVR-LSM toolbox further allows for addressing lesion volume as a nuisance variable. Prior to SVR, lesion volumes were regressed out of lesion data on a voxelwise basis and behavioral data since we found stroke volumes to be correlated with the EQ-5D. We applied an epsilon-SVR using a non-linear, radial basis function kernel analogous to the original publication found to be valid in comparable stroke imaging datasets^25^. A 20-fold cross-validated, Bayesian optimization with 200 iterations as implemented in Matlab (bayesopt) was applied to establish the optimal SVR hyperparameters related to our dataset. SVR was subsequently run using optimized hyperparameters with 10,000 permutations and a voxelwise threshold of p < 0.005 to detect voxels with significant association with clinical behavior. Co-localized voxels were then grouped into clusters and an additional family wise error (FWE) correction of p < 0.05 was applied. In an exploratory approach, we also report the presence and location of clusters consisting of > 50 contiguous voxels, but not meeting full FWE correction. Anatomical location of all clusters was determined according to the Harvard–Oxford Cortical and Subcortical Structural Atlas and the Juelich Histological Atlas^26,27^. In addition, we explored the anatomical location of brain areas with significant lesion-symptom associations in relation to white matter fiber bundles. Therefore, we mapped clusters of significant voxels resulting from SVR-LSM onto a white matter atlas. We applied the Tractotron toolbox (standalone version) of the BCB Toolkit (Foulon et al.^11^; http://www.toolkit.bcblab.com), which comprises a probabilistic white matter tractography atlas derived from 47 healthy individuals. For each fibre tract, we quantified the certainty that it intersects a cluster of voxels by extracting, for each voxel in the cluster, the probability of it belonging to the fibre tract, and then taking the maximum over all voxels in the cluster^11,28,29^. For our analysis, we report fiber tracts that intersect significant clusters after SVR-LSM with a certainty > 90%.

## Supplementary Information

Supplementary Information.

## Abbreviations

DWI: Diffusion-weighted imaging
EQ-5D: European Quality of Life 5 Dimensions
FLAIR: Fluid-attenuated inversion recovery
FEW: Familywise error correction
IQR: Inter-quartile range
LSM: Lesion-symptom mapping
MCA: Middle cerebral artery
MNI: Montreal Neurological Institute
mRS: Modified Rankin Scale
NIHSS: National Institutes of Health Stroke Scale
PROMs: Patient reported outcome measures
QoL: Quality of life
SD: Standard deviation
SVR: Support vector regression
