# Supplementary material for: Influence of stroke infarct location on quality of life assessed in a multivariate lesion-symptom mapping study
Source: Sci Rep. 2021 Jun 29;11:13490. doi: 10.1038/s41598-021-92865-x (PMC8241844; doi:10.1038/s41598-021-92865-x)
Supplement: Supplementary file 1 — Supplementary Information. [file 41598_2021_92865_MOESM1_ESM.docx]

**Influence of Stroke Infarct Location on Quality of Life assessed in a multivariate lesion-symptom mapping study**

Alina Königsberg, MD^1*^, Andrew T. DeMarco, PhD^2^, Carola Mayer^1^, Anke Wouters, PhD^16^, Eckhard Schlemm^1^, MBBS, PhD, Martin Ebinger, MD^6,7^ , Tae-Hee Cho, MD^8^, Matthias Endres, MD^6,9^, Jochen B. Fiebach, MD^6^, Jens Fiehler, MD^10^, Ivana Galinovic, MD^6^, Josep Puig, MD^11^, Vincent Thijs, MD^12,13^, Robin Lemmens, MD^3,4,5^, Keith W. Muir, MD^14^, Norbert Nighoghossian, MD^8^, Salvador Pedraza, MD^11^, Claus Z. Simonsen, MD, PhD^15^, Christian Gerloff, MD^1^, Götz Thomalla, MD^1^, Bastian Cheng, MD^1^

^1^ Klinik und Poliklinik für Neurologie, Kopf- und Neurozentrum, University Medical Center Hamburg-Eppendorf, Martinistr. 52, 20246 Hamburg, Germany

^2^ Department of Rehabilitation Medicine, Georgetown University, Washington, District of Columbia

^3^ Department of Neurology, University Hospitals Leuven, Herestraat 49, 3000 Leuven, Belgium

^4^ KU Leuven – University of Leuven, Department of Neurosciences, Experimental Neurology, Oude Markt 13, bus 5005, 3000 Leuven, Belgium

^5^ VIB, Center for Brain & Disease Research, Laboratory of Neurobiology, Campus Gasthuisberg, Herestraat 49, bus 602, 3000 Leuven, Belgium

^6^ Centrum für Schlaganfallforschung Berlin (CSB), Charité - Universitätsmedizin Berlin, Campus Mitte, Charitéplatz 1, 10117 Berlin, Germany

^7^ Neurologie der Rehaklinik Medical Park Humboldtmühle, An der Mühle 2-9, 13507 Berlin, Germany

^8^ Department of Stroke Medicine, Université Claude Bernard Lyon 1, CREATIS CNRS UMR 5220-INSERM U1206, INSA-Lyon; Hospices Civils de Lyon, Lyon, France

^9^ Klinik und Hochschulambulanz für Neurologie, Charité-Universitätsmedizin Berlin, Campus Mitte, Charitéplatz 1, 10117 Berlin, Germany

^10^ Department of Diagnostic and Interventional Neuroradiology, University Medical Center Hamburg-Eppendorf, Martinistr. 52, 20246 Hamburg, Germany

^11^ Department of Radiology, Institut de Diagnostic per la Image (IDI), Hospital Dr Josep Trueta, Institut d’Investigació Biomèdica de Girona (IDIBGI), Parc Hospitalari Martí i Julià de Salt - Edifici M2, 17190 Salt, Girona, Spain

^12^ Stroke Division, Florey Institute of Neuroscience and Mental Health, University of Melbourne, 245 Burgundy Street, Heidelberg, VIC 3084, Victoria, Australia

^13^ Austin Health, Department of Neurology, 145 Studley Road, Heidelberg, VIC 3084, Australia

^14^ Institute of Neuroscience & Psychology, University of Glasgow, Queen Elizabeth University Hospital, Glasgow, UK

^15^ Department of Neurology, Aarhus University Hospital, 8200 Aarhus, Denmark

^16^ Neurology, Amsterdam University Medical Centers, AMC, Amsterdam

***Corresponding Author**

Alina Königsberg

Klinik und Poliklinik für Neurologie

University Medical Center Hamburg-Eppendorf

Martinistraße 52, 20246 Hamburg, Germany

Telephone: +49 (0) 15222826804

E-mail: [a.koenigsberg@uke.de](mailto:a.koenigsberg@uke.de)

**Supplement**

|  | Mobility | Self-Care | Usual Activities | Pain/ Discomfort | Anxiety/ Depression |
| --- | --- | --- | --- | --- | --- |
| Level 1  ( 1 point) | I have no problems in walking about. | I have no problems with self-care. | I have no problems with performing my usual activities. | I have no pain or discomfort. | I am not anxious or depressed. |
| Level 2  ( 2 points) | I have some problems in walking about. | I have some problems with washing or dressing myself. | I have some problems with performing my usual activities. | I have moderate pain or discomfort. | I am moderately anxious or depressed. |
| Level 3  ( 3 points) | I am confined to bed. | I am unable to wash or dress myself. | I am unable to perform my usual activities. | I have extreme pain or discomfort. | I am extremely anxious or depressed. |

Table S1: Detailed items of the EQ-5D-3-Level score

| Variable | Cost/ Box Constraint | Sigma/Kernel Scale | Epsilon | Prediction accuracy | Pattern reproducibility index |
| --- | --- | --- | --- | --- | --- |
| EQ5D sum | 134.19 | 2.71 | 0.39 | 0.41 | 0.85 |

Table S2: Model parameters and quality reports for SVR LSM. Optimization of hyperparameters was performed via resubstitution loss and Bayesian Optimization with 200 iterations. A 20 fold cross-validation was used. Prediction accuracy is defined as the mean density of correlation coefficients between predicted scores and training scores across 10 replications of a 5-fold cross-validated model (cf. fig. S2, density plot on the left side). The pattern reproducibility index is calculated as a density of voxelwise correlation coefficients computed pairwise between 10 replicates of SVR-β maps, each generated using a random 80% of observations (cf. fig. S2, density plot on the right side).


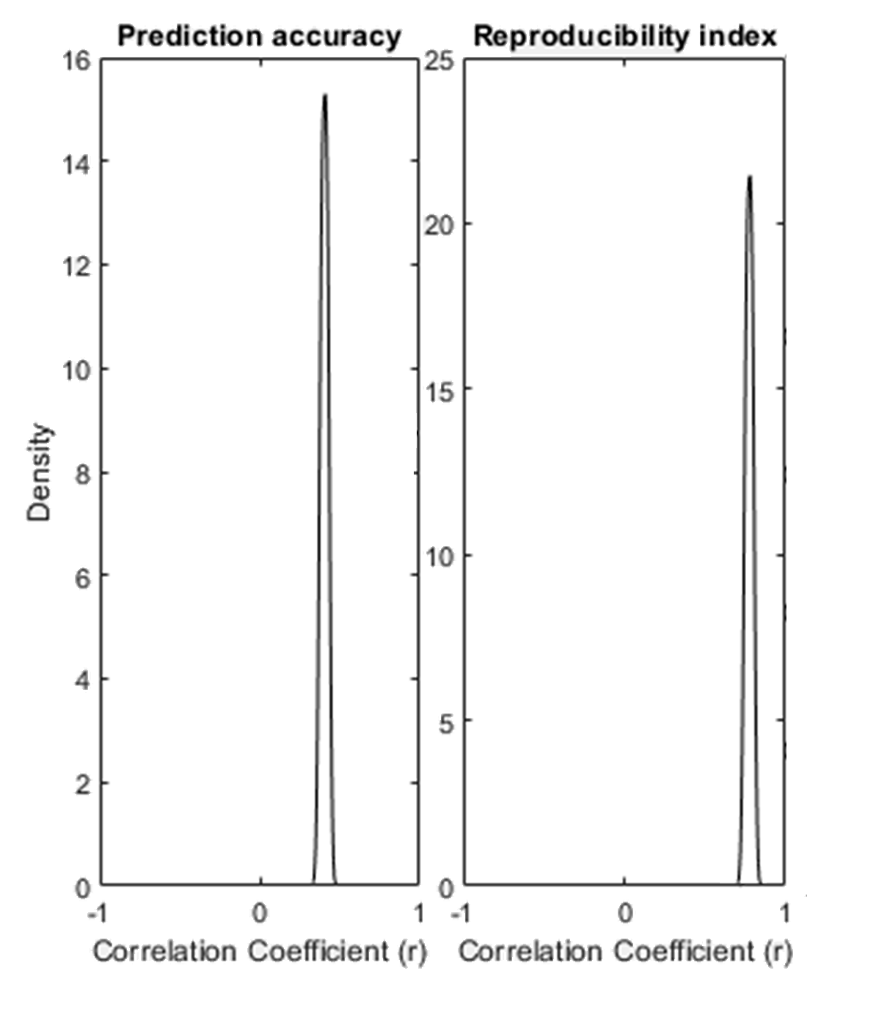


Figure S1: Quality reports for SVR LSM (see also table S2).
